# Transplanted human thymus slices induce and support T‐cell development in mice after cryopreservation

**DOI:** 10.1002/eji.201747193

**Published:** 2018-02-01

**Authors:** Susan Ross, Melissa Cheung, Ching‐In Lau, Neil Sebire, Michael Burch, Peter Kilbride, Barry Fuller, G. John Morris, E Graham Davies, Tessa Crompton

**Affiliations:** ^1^ UCL Great Ormond Street Institute of Child Health London UK; ^2^ Great Ormond Street Hospital London UK; ^3^ UCL Division of Surgery and Interventional Science Royal Free Hospital Campus London UK; ^4^ Asymptote Ltd Sovereign House, Vision Park Cambridge UK

**Keywords:** Clinical immunology, Cryopreservation, Thymopoiesis, Thymus transplantation, Transplantation

## Abstract

Nude mouse human thymus transplant model: Fresh or cryopreserved and thawed human thymus slices were transplanted subcutaneously into recipient nude mice. Nude mice subsequently produced mouse CD3^+^CD4^+^ T‐cells.

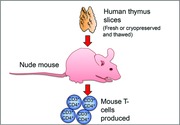

Here we show that slices of human thymus tissue that have been frozen and thawed can induce and support T‐cell development when transplanted into nude mice.

Infants born without a thymus require urgent treatment to reconstitute T‐cell immunity 1. Thymus tissue is removed from infants during cardiac surgery, to allow access to the heart. This discarded thymus tissue can be transplanted into athymic infants 2, 3, 4. Slices of thymus tissue are transplanted into the thigh, after a 2‐ to 3‐week culture period to deplete thymocytes. This procedure is life‐saving, but recipients have low T‐cell counts, and may develop autoimmunity. It is not possible to attempt to MHC‐match transplants between donor and recipient because of the urgency of performing the procedure 2, 3, 5. As delays in thymus transplantation could be life‐threatening, the procedure would be improved if it were possible to freeze thymus slices for transplantation. Cryopreservation would also open up the possibility of partial MHC‐matching.

We therefore decided to use a nude mouse model of human thymus transplantation 6 to test if slices of human thymus tissue that have been frozen and thawed could still induce and support T‐cell development. The slices that are transplanted into patients are approximately 1mm thick 2, 3, 4, 5. Cryopreservation of such thick pieces of tissue on the scale required for clinical use is technically challenging because of heat transfer issues, choice of cryoprotectants, cooling protocols and the control of ice nucleation, each of which are complex events that impact on viability of thawed tissue 7, 8, 12. We therefore froze the slices in a large‐scale cryocooler‐based control‐rate freezer that can process the larger volumes of tissue required for transplantation of thymus slices into patients, and which is compliant with good manufacturing process (GMP)‐cryopreservation 7. On thawing, slices were cultured for 24 h before histological analysis. Tissue architecture of the slices appeared similar to control fresh human thymus slices, with most of the slices appearing to be normal viable tissue (Fig. 1A). There were, however some patchy areas of autolysis comprising an estimated 5–25% of the sections studied (data not shown). Staining with a pan‐cytokeratin antibody revealed a normal lacy pattern, in both slices that had been frozen and control slices, again suggesting that the freezing process had not seriously disrupted the architecture of the epithelial component of the thymus (Fig. 1B).

**Figure 1:**
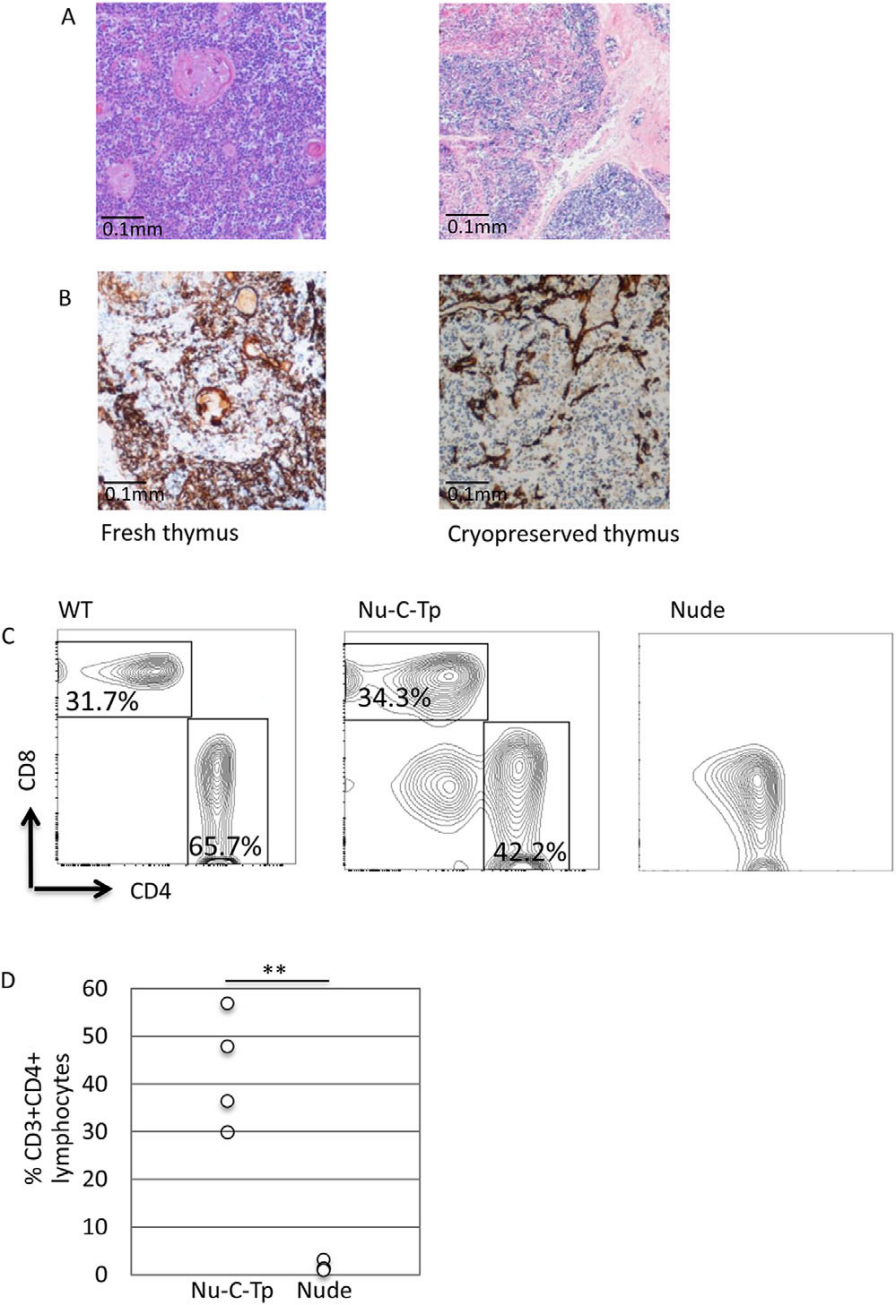
Human thymus slices that have been cryopreserved can induce and support T‐cell development in nude mice. (A) Histology of fresh human thymus and human thymus slices that have been frozen, thawed, and cultured for 24 h (hematoxylin and eosin staining, ×40 magnification). (B) Pan‐cytokeratin staining on slices as in (A). Scale bar: 0.1 mm data are representative of nine independent experiments (different thymuses) with each thymus examined in three sections. (C and D) T‐cell populations in lymph node from nude mice at 12 weeks after transplantation with cryopreserved human thymus (Nu‐C‐Tp), compared to wildtype (WT) and to untransplanted nude (Nude). Cells were analyzed by flow cytometry as described 13, gating on cells that stained negative for anti‐mouse CD11c‐PE, anti‐mouse CD11b‐PE, anti‐mouse CD45R‐PE, anti‐mouse γδ T‐cell receptor‐PE and anti‐mouse NK1.1‐PE (C) Contour plots show anti‐mouse CD4‐PerCPCy5.5 and anti‐mouse CD8‐APC staining on lymph node cells from WT control (left‐hand plot), nude transplanted with cryopreserved human thymus slices (Nu‐C‐Tp, middle plot) and control non‐transplanted nude (Nude, right‐hand plot). The percentage of CD8 T‐cells and CD4 T‐cells is given for each region shown. (D) The scatter plot shows the percentage of CD3^+^‐FITC CD4^+^‐PerCPCy5.5 T‐cells from the lymph nodes of independent transplanted nude mice (Nu‐C‐Tp, *n* = 4) and control (non‐transplanted) nude mice (Nude, *n* = 4). Detailed gating strategy is reported in the Supporting information. Each data point represents an individual mouse transplanted with slices from a different human thymus. The difference in mean between the two groups of mice was significant by Student's *t*‐test (*p* = 0.005, **p≤0.01). Data are representative of four independent experiments.

Thawed slices were cultured for 24 h before transplantation into athymic nude mice, as described previously 6. The transplanted human thymus slices were able to induce and support mouse T‐cell development, with the same kinetics as human thymus slices that had not been cryopreserved 6. Peripheral CD4^+^ and CD8^+^ T‐cells were observed 12 weeks after transplantation (Fig. 1C and D). These CD4^+^ and CD8^+^ cells in the transplanted animals stained positive for CD3, confirming they were T‐cells (Fig. 2A). There were no significant differences in the number of CD4^+^CD3^+^ cells or between CD4^+^CD25^−^ and CD4^+^CD25^+^ cells in lymph nodes between mice transplanted with fresh thymus slices and slices that had been frozen and thawed (Fig. 2B and 6). All three experimental groups contained similar numbers of CD8^+^CD3^+^ and γδ^+^CD3^+^ cells (Fig. 2B), consistent with their extrathymic differentiation in nude mice 9, 10. As expected 6 we detected no donor (human) T cells in the transplanted mice (Fig. 2C).

**Figure 2:**
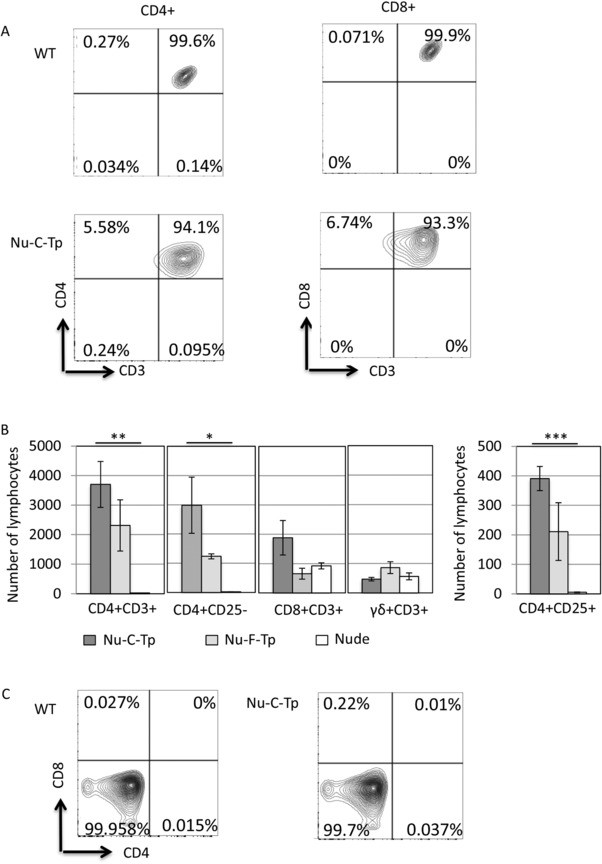
Mouse T‐cells are CD3 positive in nude mice transplanted with cryopreserved human thymus and human T‐cells were not detected. (A) T‐cell populations in lymph node from nude mice at 12 weeks after transplantation with cryopreserved human thymus (Nu‐C‐Tp), compared to wild‐type (WT). Cells were analyzed by flow cytometry, gating on cells that stained negative for anti‐mouse CD11c‐PE, anti‐mouse CD11b‐PE, anti‐mouse CD45R‐PE, anti‐mouse γδ T‐cell receptor‐PE and anti‐mouse NK1.1‐PE. Contour plots show, for left‐hand column, anti‐mouse CD4‐PerCPCy5.5 versus anti‐mouse CD3‐FITC staining, gated on CD4‐PerCPCy5.5 positive, and for right‐hand column, anti‐mouse CD8‐APC versus anti‐mouse CD3‐FITC staining, gated on CD8‐positive, for control WT (upper panel) and nude transplanted with cryopreserved human thymus slices (Nu‐C‐Tp, lower panel). The percentage of cells in each quadrant is given. (B) T‐cell populations in lymph node from nude mice at 12 weeks after transplantation with cryopreserved human thymus (Nu‐C‐Tp) or human thymus that has not been cryopreserved (Nu‐F‐Tp), compared to nude control. Bar graphs show mean number of T‐cells ± SEM per 100 000 cells recovered from lymph nodes, *n* = 3 with exception of transplanted cryopreserved thymus (Nu‐C‐Tp) CD3^+^CD4^+^ and CD3^+^CD8^+^ *n* = 4 and transplanted fresh thymus (Nu‐F‐Tp) CD4^+^CD25^−^ and CD4^+^CD25^+^ *n* = 2. Statistical significance using the Student's *t*‐test was assessed between the nude mice transplanted with cryopreserved thymus and control non‐transplanted nude mice, CD3^+^CD4^+^ *p* = 0.005. **p≤0.01, CD4^+^CD25^−^ *p* = 0.018, *p≤0.05 and CD4^+^CD25^+^ *p* = 0.0002 ***p≤0.001. Data are representative of three independent experiments. (C) T‐cell populations in lymph node from nude mice 12 weeks after transplantation with cryopreserved human thymus (Nu‐C‐Tp), compared to WT. Contour plots show staining against human‐CD8‐APC and human‐CD4‐FITC on lymph node cells from WT (left‐hand plot) and nude transplanted with cryopreserved human thymus slices (Nu‐C‐Tp, right‐hand plot). Data are representative of four independent experiments with three mice per condition. The percentage of cells in each quadrant is given.

In conclusion, these animal experiments show that slices of human thymus can still induce and support T‐cell development after cryopreservation. The tissue that has been frozen behaves in a comparable manner to fresh human thymus slices in our animal model 6. This opens up the possibility of freezing thymus slices for transplantation into human patients, thus reducing delays in immune reconstitution for severely immunodeficient infants while waiting for a suitable thymus‐donor, and enabling better MHC‐matching. As thymocytes are particularly susceptible to induction of apoptosis 11, freezing tissue slices may have the added advantage of reducing the need for the 2‐ to 3‐week tissue culture period to deplete the tissue of donor T‐cells.

## Author contributions

S.R., M.C., C.I.L., N.S., and E.G.D. carried out experimental work; M.B., T.C., and E.G.D. obtained funding; P.K., B.F., and G.J.M. provided expertise in cryopreservation; S.R. and T.C. wrote manuscript.

## Conflict of interest

The authors declare no financial or commercial conflict of interest.

**Abbreviations**
Nu‐C‐Tp: nude mouse cryopreserved human thymus transplant
Nu‐F‐Tp: nude mouse fresh thymus transplant
WT: wild type

## Supporting information

Supporting Information

Peer review correspondence
