# Supplementary material for: Transplanted human thymus slices induce and support T‐cell development in mice after cryopreservation
Source: Eur J Immunol. 2018 Feb 1;48(4):716–9. doi: 10.1002/eji.201747193 (PMC5947170; doi:10.1002/eji.201747193)
Supplement: Supplementary file 1 — Supporting Information [file EJI-48-716-s001.pdf]

European Journal of Immunology

Supporting Information

for

DOI 10.1002/eji.201747193

Susan Ross, Melissa Cheung, Ching-In Lau, Neil Sebire, Michael Burch, Peter Kilbride, Barry Fuller, G. John Morris, E Graham Davies and Tessa Crompton

Transplanted human thymus slices induce and support T-cell development in mice after cryopreservation

© 2018 The Authors. *European Journal of Immunology* published by WILEY-VCH Verlag GmbH & Co. KGaA, Weinheim.

Supporting Information

Materials and methods

Cryopreservation and defrosting of human thymus tissue

Thymus tissue sections were prepared and the slices were mounted on nitrocellulose filters (Millipore) on medium (Ham's F10 supplemented with Hepes, L Glutamine and 10% Fetal bovine serum) as described previously [4]. For freezing, we used a slow cooling protocol based on cryopreservation of ovarian tissue fragments [8, 12]. The nitrocellulose filters with thymus slices on them were rolled into a loose cylindrical shape and inserted into 1.8ml cryovials (Nunc), with or without a single glass bead or autonucleation beads (IceStart autonucleation beads (Asymptote, Cambridge, UK)). The cryovial was filled with Cryostor CS10 (Sigma). Cryovials were transferred into a controlled rate freezer (VIAFreeze, Asymptote, UK) pre-cooled to 4°C. The freezing program was: -0.3°C per minute to -10°C, dwell at -10°C for 10 minutes, -0.3°C per minute to -40°C, -1°C per minute to -80°C. The hold step at -10°C allowed time for ice nucleation and for dissipation of latent heat of ice formation, which can otherwise disrupt control of cooling. Samples were then held at -80°C until transfer to liquid nitrogen. Transplants were performed within 5 weeks of freezing.

To defrost, cryovials were placed in a 37°C water bath until ice had disappeared, then the thymus slices and underlying filters were washed twice in medium for 10 minutes. Slices were cultured on filters in thymus culture medium for 24 hours before histological analysis or transplantation into mice. The study

was conducted with institutional ethical approval, with written informed consent, according to the Declaration of Helsinki principles.

Mice

C57Bl/6 (B6) were purchased from Harlan (UK) and nude (B6.Cg-Foxn1nu/J) mice from Jackson Labs (Bar Harbour, ME). Animals were housed in individually ventilated cages at UCL and underwent procedures under UK Home Office regulations.

Xenografts

Human thymus tissue was subcutaneously injected in phosphate-buffered saline into the scruff of mice, as described previously [6].

Cell preparation

Mouse tissue was teased through a cell strainer into PBS + 5% FBS + 0.01% azide in preparation for flow cytometry.

Flow cytometry

Cells were stained as described [13] with fluorochrome-conjugated antibodies (eBioscience, Hatfield, UK or BD Pharmingen, Oxford, UK) in PBS + 5% FBS + 0.01% azide for 15 minutes at room temperature, and washed and analyzed by flow cytometry (Instrument: Accuri C6 flow cytometer, BD; software: Accuri C6, BD, and Flowjo, Treestar, Ashland, OR). The gating strategy is shown in supplementary figure 1.

Histology

Human thymus tissue was fixed in formalin and then paraffin wax embedded. Tissue was sectioned (4μm) and stained as previously described using H&E and anti-pan cytokeratin antibody (AE1/AE3, Dako, Glostrup, Denmark) [3, 6]. To

assess extent of autolytic changes, sections of the tissue were viewed under low power and the proportion of the total of the slice showing changes was estimated.

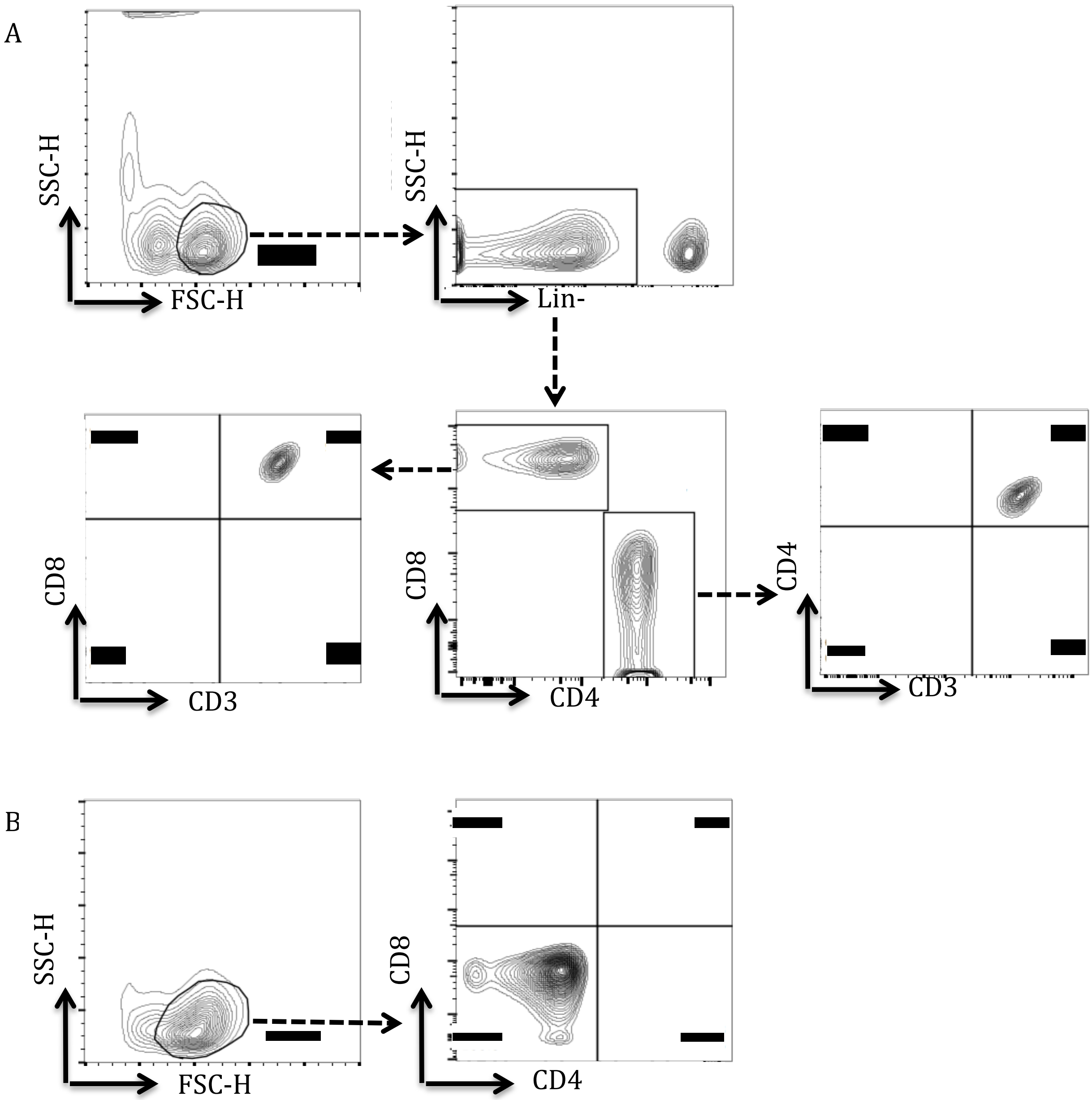

Supporting information Figure 1: Gating strategy for flow cytometry analysis.
For analysis of mouse CD4+CD3+ and CD8+CD3+ T-cells: live lymphocytes were gated by FSC and SSC, and then gated as negative for anti-mouse CD11c, anti-mouse CD11b, anti-mouse CD45R, anti-mouse γδ T-cell receptor and anti-mouse (Lin-), to anlayse CD4+CD8- and CD4-CD8+ cells for expression of cell surface CD3. (B) Analysis of human CD8+ and CD4+ cells in transplanted and control mice. Initially, mouse lymph node cells were stained for anti-human CD4 and anti-human CD8, a live gate was drawn by FSC, SSC profile, and expression of human-CD4 against human-CD8 cells plotted.
