# Supplementary material for: Transplanted human thymus slices induce and support T‐cell development in mice after cryopreservation
Source: Eur J Immunol. 2018 Feb 1;48(4):716–9. doi: 10.1002/eji.201747193 (PMC5947170; doi:10.1002/eji.201747193)
Supplement: Supplementary file 2 — Peer review correspondence [file EJI-48-716-s002.pdf]

European Journal of Immunology

Transplanted human thymus slices induce and support T-cell development in mice after cryopreservation

Susan Ross, Melissa Cheung, Ching-In Lau, Neil Sebire, Michael Burch, Peter Kilbride, Barry Fuller, G. John Morris, E Graham Davies andTessa Crompton

Correspondence: Dr. Tessa Crompton, UCL Great Ormond Street Institute of Child Health, London, UK

Review Timeline:	Submission date:	22-Jun-2017
	First Editorial decision:	07-Aug-2017
	Revision received:	20-Nov-2017
	Accepted:	04.01.2018

Handling Executive Committee member: Prof. Lucienne Chatenoud

Please note that the correspondence below does not include the standard editorial instructions regarding preparation and submission of revised manuscripts, only the scientific revisions requested and addressed.

First Editorial Decision
07-Aug-2017

Dear Dr. Crompton,

We sincerely apologize for the delay in the peer review of your Manuscript ID eji.201747193 entitled "Transplanted human thymus slices induce and support T-cell development in mice after cryopreservation" which you submitted to the European Journal of Immunology. There was a severe delay in receiving one of the reports. The comments of the referees are included at the bottom of this letter.

A revised version of your manuscript that takes into account the comments of the referees will be reconsidered for publication. You will see that Referee 1 requested additional experiments/data (absolute numbers of various cell subsets, graft survival and statistical analysis), which are essential. Should you disagree with any of the referees´concerns, you should address this in your point-by-point response and provide solid scientific reasons for why you will not make the requested changes.

You should also pay close attention to the editorial comments included below. **In particular, please edit

your figure legends to follow Journal standards as outlined in the editorial comments. Failure to do this will result in delays in the re-review process.**

Please note that submitting a revision of your manuscript does not guarantee eventual acceptance, and that your revision will be re-reviewed by the referees before a decision is rendered.

If the revision of the paper is expected to take more than three months, please inform the editorial office. Revisions taking longer than six months may be assessed by new referees to ensure the relevance and timeliness of the data.

Once again, thank you for submitting your manuscript to European Journal of Immunology and we look forward to receiving your revision.

Yours sincerely,
Nadja Bakocevic

On behalf of
Dr. Lucienne Chatenoud

Dr. Nadja Bakocevic
Editorial Office
European Journal of Immunology
e-mail: ejied@wiley.com
www.eji-journal.eu

****

Reviewer: 1

Comments to the Author
The manuscript by Roos et al describes T cell production after the transplantation of freshly isolated or cryopreserved human thymus into nude mice. This is important because human thymus are not frequently available and being able to use cryopreserved tissue would greatly improve the availability of a transplantable tissue that could then be partially MHC matched. It is shown by flow cytometry analysis that the transplantation of human thymic slices results in the production of mouse T cells expressing CD4, CD8 and CD3. No other subsets and no numbers of cells produced are indicated. Although acknowledging the clinical potential of the observations it is strictly necessary to have numbers of cells and to ascertain that

the same subsets produced with fresh tissue are also produced with frozen slices. The numbers of Î³Î´+, NK T and Tregs as well as those of CD8 and CD4+CD25- should be included. Another important issue that should be studied is the survival of the graft and the capacity in time to produce T cells, comparing fresh with frozen tissue. We also need to know how many grafts were performed and have a statistical analysis on the number of cells obtained.

Reviewer: 2

Comments to the Author
Ross and colleagues describe the cryopreservation and subsequent thawing and implantation of human thymus tissue into a mouse model, and demonstrate that thymic architecture is largely preserved despite freeze-thawing, and that cryopreserved tissue is able to support T-lymphocyte development.

The article is well-wriiten and clear, the diagrams are relevant and support the conclusions and are adequately labelled.

I have only a couple of minor comments.

Are the authors able to give an indication as to whether there is a maximum time of freezing, beyond which there is a deterioration in tissue viability?
Page 2, line 12 -´Infants´would be better than ´babies´.

First Revision – authors' response
20-Nov-2017

Point-by-point response

Reviewer: 1

Comments to the Author
The manuscript by Roos et al describes T cell production after the transplantation of freshly isolated or cryopreserved human thymus into nude mice. This is important because human thymus are not frequently available and being able to use cryopreserved tissue would greatly improve the availability of a transplantable tissue that could then be partially MHC matched. It is shown by flow cytometry analysis that the transplantation of human thymic slices results in the production of mouse T cells expressing CD4, CD8 and CD3. No other subsets and no numbers of cells produced are indicated. Although acknowledging the clinical potential of the observations it is strictly necessary to have numbers of cells and to ascertain that the same subsets produced with fresh tissue are also produced with frozen slices.

European Journal of Immunology

The numbers of γδ+, NK T and Tregs as well as those of CD8 and CD4+CD25- should be included.

RESPONSE: We have added a comparison showing the actual number of cells produced between transplantation of frozen and fresh tissue, for CD4+CD3+, and subdividing the CD4+ population by expression of CD25 (CD4+CD25-, CD4+CD25+), and CD8+CD3+ and CD3+γδ+ (highlighted in yellow). This is shown in Figure 2. We did not include data for the NK lineage (either NK cells or NKT cells), because, as others have also reported, we found that the host nude my already contained NK cells, and in comparison between WT, we did not find that the host C57BL/6 nudes had reduced numbers.
Our study is a technical comment only, with the sole aim of providing proof-of-principle that it is possible to successfully freeze and thaw these relatively think slices of tissue. We therefore did not carry out a detailed analysis of T-cell development and T-cell subpopulations in the transplanted mice, but focused on the appearance of the CD4+CD3+ population, which is so key to the reconstitution of adaptive immunity in complete DiGeorge syndrome patients.

Another important issue that should be studied is the survival of the graft and the capacity in time to produce T cells, comparing fresh with frozen tissue.

RESPONSE: Our study is a short technical comment, to examine whether or not human thymus slices that have been frozen and thawed can support T-cell development. We have not attempted to determine how long the graft survives or its capacity to produce T-cells through time. In deed, this information is not known for fresh human thymus slices, and it is difficult to investigate effectively in a mouse model, because of the relatively short lifespan of mice compared to humans. In the case of athymic infants, the first and most urgent requirement of transplanted thymus is to reconstitute the peripheral CD4 T-cell population, and if a graft did not survive well, the child should still benefit from T-cell mediated immunity, in the same way that thymectomised cardiac surgery patients sustain T-cell immunity for many years after thymectomy.

We also need to know how many grafts were performed and have a statistical analysis on the number of cells obtained.

RESPONSE:The number of grafts performed and the statistical analysis are provided in the figures and figure legends.

Reviewer: 2

Comments to the Author
Ross and colleagues describe the cryopreservation and subsequent thawing and implantation of human thymus tissue into a mouse model, and demonstrate that thymic architecture is largely preserved despite freeze-thawing, and that cryopreserved tissue is able to support T-lymphocyte development.

European Journal of Immunology

The article is well-written and clear, the diagrams are relevant and support the conclusions and are adequately labelled.

I have only a couple of minor comments.

Are the authors able to give an indication as to whether there is a maximum time of freezing, beyond which there is a deterioration in tissue viability?

RESPONSE: Our technical comment focuses on whether it is possible to freeze and thaw slices of human thymus tissue (which are around 1mm thick) and whether they can still support T-cell development after freezing and thawing. As such, we did not assess how long we can store such slices of thymus tissue after freezing, and the longest we stored slices and then used them to reconstitute T-cell development in mice was 5 weeks. We have added a sentence to the methods to state the length of storage (Materials and Methods, Page 8, highlighted in yellow).

Page 2, line 12 -'Infants' would be better than 'babies'.

RESPONSE: We have changed 'babies' to 'Infants' (highlighted in yellow).

Second Editorial Decision
30-Nov-2017

Dear Dr. Crompton,

It is a pleasure to provisionally accept your manuscript entitled "Transplanted human thymus slices induce and support T-cell development in mice after cryopreservation" for publication in the European Journal of Immunology. For final acceptance, please follow the instructions below and return the requested items as soon as possible as we cannot process your manuscript further until all items listed below are dealt with.

Please note that EJI articles are now published online a few days after final acceptance (see Accepted Articles: http://onlinelibrary.wiley.com/journal/10.1002/(ISSN)1521-4141/accepted). The files used for the Accepted Articles are the final files and information supplied by you in Manuscript Central. You should therefore check that all the information (including author names) is correct as changes will NOT be permitted until the proofs stage.

We look forward to hearing from you and thank you for submitting your manuscript to the European Journal of Immunology.

Yours sincerely,

Marta Vuerich

on behalf of

Dr. Lucienne Chatenoud

Dr. Marta Vuerich

Editorial Office

European Journal of Immunology

e-mail: ejied@wiley.com

www.eji-journal.eu
